# Synaptic and brain-expressed gene sets relate to the shared genetic risk across five psychiatric disorders

**DOI:** 10.1017/S0033291719001776

**Published:** 2020-07

**Authors:** Anke R. Hammerschlag, Christiaan A. de Leeuw, Christel M. Middeldorp, Tinca J. C. Polderman

**Affiliations:** 1 Department of Complex Trait Genetics, Center for Neurogenomics and Cognitive Research, Amsterdam Neuroscience, Vrije Universiteit Amsterdam, Amsterdam, The Netherlands; 2 Child Health Research Centre, the University of Queensland, Brisbane, QLD, Australia; 3 Department of Biological Psychology, Amsterdam Public Health, Vrije Universiteit Amsterdam, Amsterdam, The Netherlands; 4 Child and Youth Mental Health Service, Children's Health Queensland Hospital and Health Service, Brisbane, QLD, Australia

**Keywords:** Attention-deficit/hyperactivity disorder, biological pathways, bipolar disorder, comorbidity, gene-set analysis, major depressive disorder, schizophrenia

## Abstract

**Background:**

Mounting evidence shows genetic overlap between multiple psychiatric disorders. However, the biological underpinnings of shared risk for psychiatric disorders are not yet fully uncovered. The identification of underlying biological mechanisms is crucial for the progress in the treatment of these disorders.

**Methods:**

We applied gene-set analysis including 7372 gene sets, and 53 tissue-type specific gene-expression profiles to identify sets of genes that are involved in the etiology of multiple psychiatric disorders. We included genome-wide meta-association data of the five psychiatric disorders schizophrenia, bipolar disorder, major depressive disorder, autism spectrum disorder, and attention-deficit/hyperactivity disorder. The total dataset contained 159 219 cases and 262 481 controls.

**Results:**

We identified 19 gene sets that were significantly associated with the five psychiatric disorders combined, of which we excluded five sets because their associations were likely driven by schizophrenia only. Conditional analyses showed independent effects of several gene sets that in particular relate to the synapse. In addition, we found independent effects of gene expression levels in the cerebellum and frontal cortex.

**Conclusions:**

We obtained novel evidence for shared biological mechanisms that act across psychiatric disorders and we showed that several gene sets that have been related to individual disorders play a role in a broader range of psychiatric disorders.

## Introduction

Psychiatric disorders pose an enormous burden on affected individuals, their families, and society as a whole, and insight into causal factors and successful treatment is strikingly limited (Akil *et al*., 2010). For decades, psychiatric disorders are classified based on their observed symptoms and disease course. Yet, it is debated to what extent the disorders are distinct entities as boundaries are difficult to define. Psychiatric disorders contain a strong genetic component (Polderman *et al*., 2015) and mounting evidence shows genetic overlap of common single nucleotide variants (SNPs) between multiple disorders (Cross-Disorder Group of the Psychiatric Genomics Consortium, 2013*a*, 2013*b*; Bulik-Sullivan *et al*., 2015*a*; Anttila *et al*., 2018; Schork *et al*., 2019), primarily schizophrenia (SCZ), bipolar disorder (BD), major depressive disorder (MDD), attention-deficit/hyperactivity disorder (ADHD) and autism spectrum disorder (ASD). This genetic overlap of common SNPs might be driven by biological mechanisms that are shared between multiple disorders. Insight into these shared neurobiological processes will optimize diagnoses, and subsequent targeted drug development and treatment for a broad range of disorders.

Recent genome-wide association studies (GWAS) have been very successful in identifying many genetic variants that are associated with single psychiatric disorders, such as SCZ (Pardinas *et al*., 2018) and MDD (Wray *et al*., 2018). Yet, knowledge on the biological underpinnings is still limited because the effects of individual genetic variants are extremely small and as such give little information about underlying biological mechanisms. To obtain more insight, an alternative approach is to study sets of genes that are part of the same biological pathway or functional mechanism. Given the polygenic nature of psychiatric disorders, it is likely that individual genetic variants cluster in genes that share a biological function. For individual psychiatric disorders, several of these gene sets have already been identified [e.g. calcium signaling sets for SCZ (Pardinas *et al*., 2018) and FMRP target genes for ASD (Jansen *et al*., 2017), for review see Sullivan and Posthuma (2015)], yet the biological mechanisms that drive a common liability for psychiatric disorders are still largely unknown. First insights were provided by a large gene-set study in 2015 that investigated the contribution of nearly 5000 gene sets derived from publicly available databases to the risk of five main psychiatric disorders; SCZ, BD, MDD, ADHD, and ASD (The Network and Pathway Analysis Subgroup of the Psychiatric Genomics Consortium, 2015). This study reported a role of gene sets involved in histone methylation, immune and neuronal signaling, and the synapse across SCZ, BD, and MDD. The smaller sample sizes of ASD and ADHD resulted in insufficient power to detect shared gene sets.

The current study aims to capitalize on the rapid increase in sample sizes for psychiatric GWASs that have led to a dramatic expansion in the identification of significantly associated genetic variants. Using these rich datasets we aim to extend previous research by testing a variety of plausible gene sets, both derived from publicly available databases such as KEGG, GO, BioCarta and Reactome, and gene sets composed by expert curation. The latter gene sets are excellent candidates for playing a role in multiple psychiatric disorders because they tend to be less biased towards well-studied genes, especially concerning genes active in the brain (Feldman *et al*., 2008; Rossin *et al*., 2011). Hence, expert-curated gene sets could give important additional insights. Furthermore, we investigated the role of specific tissue types using gene-expression profiles obtained from the Genotype-Tissue Expression (GTEx) Project (GTEx Consortium, 2015). In addition, we applied conditional analyses to systematically test the independence of significantly associated gene sets and tissue types. We used the most recent publicly available GWAS summary statistics from the five psychiatric disorders SCZ, BD, MDD, ADHD, and ASD. The aim of this study is to identify sets of genes that are involved in the etiology of multiple psychiatric disorders that subsequently can teach us more about associated neurobiological processes.

## Methods

### Sample

We used the most recent publicly available genome-wide meta-association results from five psychiatric disorders for which multiple genetic loci have been identified, i.e., these datasets provide sufficient statistical power to detect genetic associations. These datasets comprise SCZ (Pardinas *et al*., 2018) (40 675 cases and 64 643 controls), BD (Bipolar Disorder and Schizophrenia Working Group of the Psychiatric Genomics Consortium, 2018) (20 129 cases and 21 524 controls), MDD (Wray *et al*., 2018) (59 851 cases and 113 154 controls), ASD (Grove *et al*., 2019) (18 381 cases and 27 969 controls), and ADHD (Demontis *et al*., 2019) (20 183 cases and 35 191 controls). Summary statistics were downloaded from https://www.med.unc.edu/pgc/results-and-downloads. The total dataset contained 159 219 cases and 262 481 controls. Details of the datasets have been described previously (Pardinas *et al*., 2018; Bipolar Disorder and Schizophrenia Working Group of the Psychiatric Genomics Consortium, 2018; Wray *et al*., 2018; Demontis *et al*., 2019; Grove *et al*., 2019). All samples underwent similar quality control, imputation, and analysis steps.

### Genetic correlations

SNP heritability (*h*_SNP_^2^) and genetic correlations (*r*_g_) were calculated between the five psychiatric disorders using Linkage Disequilibrium (LD) Score regression (Bulik-Sullivan *et al*., 2015*b*) (https://github.com/bulik/ldsc). We used precomputed LD scores that were provided by LD score regression, which were calculated using the European panel of the 1000 Genomes Project. No constraining of the intercept was applied.

### Gene sets and tissue types

We included two types of gene sets: (1) derived from publicly available databases, and (2) expert-curated, i.e., genes annotated by experts in a specific research field that commonly involves an extensive experimental or methodological design and interpretation. In addition, we investigated tissue-specific gene-expression values as gene properties (i.e., continuous values for all genes). First, we selected from the molecular signature database [MsigDB v6.2 (Subramanian *et al*., 2005), http://software.broadinstitute.org/gsea/msigdb/] all Gene Ontology (GO) gene sets (*n* = 5917) and canonical pathways (*n* = 1329). The GO gene sets contain genes annotated by the same GO term and cover biological processes, cellular components, and molecular functions. The canonical pathways are representations of biological processes that are compiled by domain experts. Second, we selected 126 expert-curated gene sets that have been tested to date for one of the five disorders in studies based on whole genome approaches [i.e., GWAS, copy number variant (CNV) analysis, or whole-exome sequencing analysis]. These gene sets cover different types of expert-curated sets: sets of functionally related genes, sets of co-expressed genes, and sets representing protein complexes or networks (online Supplementary Table S1). Third, we obtained gene-expression values of 53 tissues from the GTEx project v7 (http://www.gtexportal.org/home/datasets). The expression values of all genes were Winsorized at 50 reads per kilobase of transcript per million reads mapped (RPKM) and subsequently log2 transformed with pseudocount 1 (Pers *et al*., 2016).

### Gene-set and gene-property analyses

We performed gene-set and gene-property analyses in MAGMA (de Leeuw *et al*., 2015) (http://ctg.cncr.nl/software/magma) with the GWAS summary statistics of the five psychiatric disorders as input. Genetic variants with imputation INFO score <0.8 were excluded. We first conducted a gene analysis for each disorder individually in MAGMA using the *snp-wise* = *mean* gene analysis model which is a test of mean SNP association using the sum of -log(SNP *p* value) as test statistic. We subsequently meta-analyzed the gene analysis results across the five disorders in MAGMA using an unweighted Stouffer's *Z*-transform method (Whitlock, 2005) (i.e., the weight of every disorder is one to correct for the potential effect of differences in sample size between disorders). Because the datasets of the five disorders partially overlap, we corrected for sample overlap by including a covariance matrix of the cross-trait LD score intercept (Baselmans *et al*., 2019; Jansen *et al*., 2019), estimated by LD Score regression, which is an estimate of sample overlap and phenotypic correlation (Bulik-Sullivan *et al*., 2015*b*). Of note, sample overlap does not affect the validity of the gene-set *p* values to a large extend (see online Supplementary Methods). The gene analysis merges the SNP associations within a gene into one test statistic, hence, the direction of SNP effects are not taken into account. This means that associated genes – and gene sets – across disorders can be influenced by different SNPs in each individual disorder, and that SNP effects can have different directions of effect in different disorders.

Next, we used the meta-analyzed gene associations as input for the competitive gene-set analyses of the database and expert-curated gene sets to test whether the genes in a gene set are more strongly associated across disorders than the other genes in the genome, thereby correcting for the baseline level of the association present in the dataset. This is especially important for polygenic traits like psychiatric disorders, because the polygenic background can cause an association of any random gene set of sufficient size. The associations were corrected for dependencies between genes by including a gene–gene correlation matrix (reflecting LD) in the regression model, and for confounding effects of gene size, gene density, mean minor allele count in the gene and per-gene sample size by adding these variables and their log values as covariates to the regression model. A Bonferroni correction was applied to correct for multiple testing.

A gene-property analysis was performed to test the associations between the tissue-type gene expressions and gene *p* values. Tissue-type gene expressions were corrected in a similar vein as the gene sets, i.e., for dependencies between genes and confounding effects of gene size, gene density, mean minor allele count in the gene and per-gene sample size. In addition, the associations were corrected for average expression over all tissue types by including this as a covariate in the model. A Bonferroni correction was applied to correct for the number of tissues tested.

We conducted several post hoc analyses of the gene sets and tissue types. First, to evaluate the contribution of each disorder to the cross-disorder associations, we performed the analyses of the gene sets and tissue types per disorder. Second, we performed a cross-disorder analysis in which we accounted for possible differences between disorders at the SNP level by taking the direction of SNP effects into account in a SNP *p* value based meta-analysis, correcting for overlap, in METAL (Willer *et al*., 2010). We subsequently used these results as input for the gene-based analysis followed by the gene-set and gene-property analysis. Only SNPs that were available in the summary statistics of all five disorders were included, and the weight of each disorder was set to one, corresponding to the initial meta-analysis. Third, we compared the cross-disorder results of the unweighted meta-analysis with a weighted meta-analysis using the Stouffer's weighted *Z*-transform method (Whitlock, 2005). Weighting the disorders by sample size emphasizes the hypothesis that specific biological mechanisms contribute to a common susceptibility to develop any psychiatric disorder, and hence each gene should have the same effect on the different disorders.

### Conditional analyses

Genes have multiple biological functions that often correlate across genes, which may cause confounding effects in gene-set analyses (de Leeuw *et al*., 2018). To address this issue and the overlap between the gene sets due to a hierarchical structure of the databases, we applied conditional gene-set analyses to evaluate possible redundancy between associations [stepwise method described in (de Leeuw *et al*., 2018)].

In the first step, we investigated the overlap in expression levels of the identified tissue types in a forward selection procedure. We started by selecting the tissue type with the strongest association across disorders on which the second most strongly associated tissue type was conditioned (in addition to average expression levels that were already included as a covariate in the main analysis). In every next step of this procedure, we repeated this by conditioning on those tissue types that remained nominal significantly associated in the previous steps. The remaining tissue types will reflect distinct association signals with the psychiatric disorders.

In the second step, we corrected the gene sets for general confounding of average gene expression levels and the tissue types that we identified in the previous step. In the third step, we performed the same forward selection procedure described in step 1 for the gene sets that remained nominally significant after step 2. These associations were conditioned on the tissues included in step 2 as well.

## Results

### Genetic correlations between psychiatric disorders

All disorders showed moderate to strong genetic correlations (Table 1) in line with previous findings (Bulik-Sullivan *et al*., 2015*a*; Anttila *et al*., 2018). This positive evidence of shared genetic factors substantiates the relevance of performing gene-set analysis across disorders to identify the possible biological mechanisms that could explain these shared genetic factors.

**Table 1.** Genetic correlations between five psychiatric disorders

	ADHD	ASD	BD	MDD	SCZ
ADHD	*h*_SNP_^2^ = 0.21 (0.013)				
ASD	*r*_g_ = 0.36 (0.051)	*h*_SNP_^2^ = 0.11 (0.022)			
	*p* = **1.38 × 10^−12^**				
BD	*r*_g_ = 0.12 (0.040)	*r*_g_ = 0.18 (0.052)	*h*_SNP_^2^ = 0.20 (0.010)		
	*p* = **2.90 × 10^−03^**	*p* = **5.02 × 10^−04^**			
MDD	*r*_g_ = 0.52 (0.041)	*r*_g_ = 0.41 (0.042)	*r*_g_ = 0.34 (0.033)	*h*_SNP_^2^ = 0.10 (0.0062)	
	*p* = **9.80 × 10^−36^**	*p* = **3.73 × 10^−22^**	*p* = **4.21 × 10^−2^**^5^		
SCZ	*r*_g_ = 0.16 (0.035)	*r*_g_ = 0.24 (0.039)	*r*_g_ = 0.69 (0.023)	*r*_g_ = 0.40 (0.028)	*h*_SNP_^2^ = 0.24 (0.0084)
	*p* = **5.77 × 10^−06^**	*p* = **1.09 × 10^−09^**	*p* = **2.21 × 10^−202^**	*p* = **5.28 × 10^−45^**	

SCZ, schizophrenia; BD, bipolar disorder; MDD, major depressive disorder; ASD, autism spectrum disorder; ADHD, attention-deficit/hyperactivity disorder; *h*_SNP_^2^, SNP heritability; *r*_g_, genetic correlation.

Bold *p* values are genetic correlations with *p* < 0.005 (Bonferroni corrected). Numbers in parentheses are standard errors.

### Gene sets and tissue types associated across psychiatric disorders

**Fig. 1.**
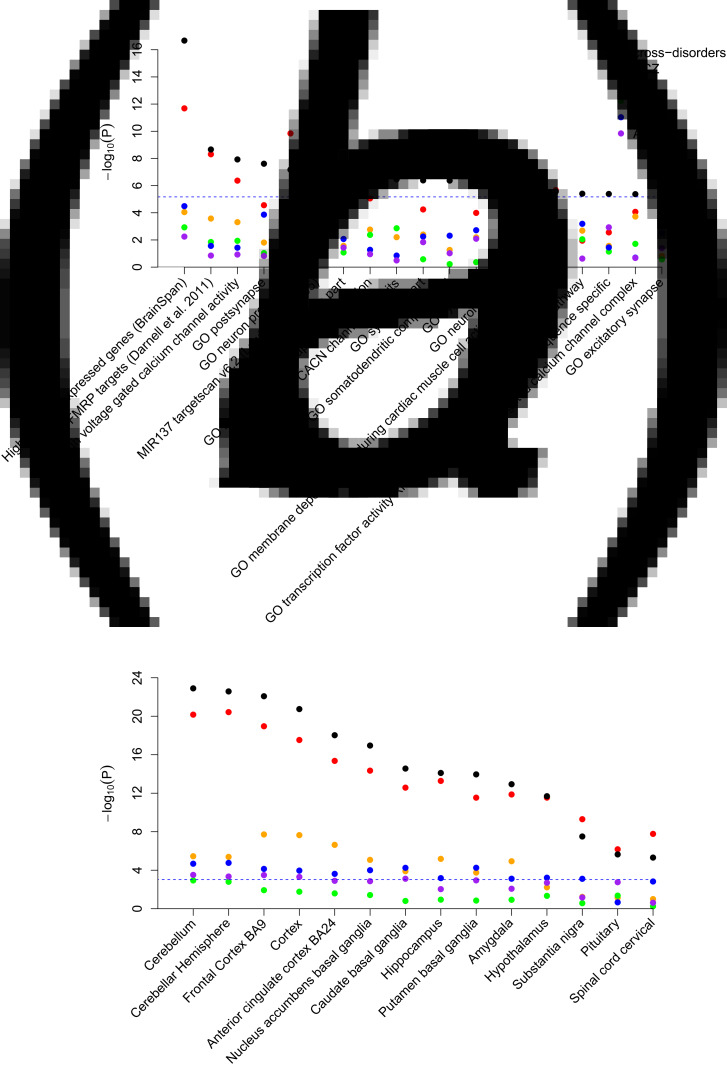
Significant gene-set and tissue-type associations across five disorders and the associations with the individual disorders. (*a*) Database and expert-curated gene sets with significant cross-disorder associations after correction for multiple testing (*α* = 6.78 × 10^−6^). The 15 GO gene sets are extracted from the Gene Ontology database. The other four gene sets are expert curated. (*b*) Tissue-type gene expression profiles with significant associations across the five disorders after correction for multiple testing (*α* = 1.48 × 10^−3^). SCZ, schizophrenia; BD, bipolar disorder; MDD, major depressive disorder; ASD, autism spectrum disorder; ADHD, attention-deficit/hyperactivity disorder.

In total, we investigated the involvement of 7372 gene sets across the five psychiatric disorders. We identified 690 gene sets that were nominal significantly associated (*α* = 0.05), of which 19 sets remained statistically significant after Bonferroni correction for multiple testing (*α* = 6.78 × 10^−6^; Fig. 1*a* and online Supplementary Table S2). A set of highly-brain-expressed genes based on BrainSpan data (Pinto *et al*., 2014) was most strongly associated across disorders (*p* = 2.11 × 10^−17^). In addition, nine gene sets related to the synapse (of which three calcium channel sets), five gene sets related to components of the neuron, targets of the Fragile X mental retardation protein (FMRP), targets of the micro RNA MIR137, a gene set related to membrane depolarization, and a gene set related to transcription were significantly associated across the five disorders. The gene-property analysis of 53 GTEx tissues resulted in significant associations of all brain tissue types (*n* = 14) after Bonferroni correction for multiple testing (*α* = 1.48 × 10^−3^; Fig. 1*b* and online Supplementary Table S3). The cerebellum was most strongly associated (*p* = 1.26 × 10^−23^), followed by the cerebellar hemispheres and the frontal cortex. None of the non-brain tissue types was significantly associated across disorders.

We compared the results of these analyses – which are based on meta-analyzing gene-based associations of the individual disorders – with a gene-set and gene-property analysis including as input the meta-analyzed SNP associations of the five disorders. Hence, the direction of the SNP effects is taken into account. The results of these analyses and the initial analyses showed strong correlations [Pearson's correlation of log_10_(*p*) = 0.71 and 0.99 for gene sets and tissue types, respectively; online Supplementary Tables S2 and S3]. Considering the significantly associated gene sets, the three calcium channel sets and the gene set involved in membrane depolarization showed equal association strengths, while the other gene sets present small to moderate reduced associations when taking the direction of SNP effects into account. This implies that the effects of SNPs related to calcium channels, or more broadly related to action potentials and depolarization, increase the risk on the different disorders in the same direction, while the effects of genetic variants related to the other identified functions partly act in opposite directions across disorders. In addition to this post hoc analysis, we performed a gene-set and gene-property analysis weighting the disorders according to sample size. These results were highly consistent with the initial unweighted analysis [Pearson's correlation of log_10_(*p*) = 0.97 and 1 for gene sets and tissue types, respectively; online Supplementary Tables S2 and S3].

To investigate the contribution of the association signal of the individual disorders to the cross-disorder association, we performed the gene-set and gene-property analyses for the individual disorders as well (Fig. 1; online Supplementary Tables S2 and S3). The contribution of SCZ was stronger compared to the other disorders, and to evaluate the effect of SCZ on the cross-disorder associations further, we tested the cross-disorder data excluding SCZ (online Supplementary Tables S2 and S3). All associations dropped substantially, yet 14 gene sets and 11 brain tissue types showed stronger associations across disorders than with SCZ only. This suggests that although the gene sets and tissues have a stronger effect on SCZ, they have effects on the other disorders as well, although considerably smaller. An effect of these gene sets in the four other disorders is further supported by a stronger association across these four disorders compared to the association with each disorder individually. Five gene sets and three brain tissue types that were significantly associated across the five disorders were more strongly associated with SCZ compared to the cross-disorder association. Since we cannot exclude that solely SCZ might drive these cross-disorder associations, we excluded these gene sets from further investigation in the conditional analyses.

### Overlap between identified gene sets and tissue types

The gene sets that were significantly associated across the five disorders are not independent as genes considerably overlap between the sets (Table 2). In addition, gene expression levels correlate between tissue types. To test the independence of the significantly associated gene sets and tissue types – excluding the five gene sets and three tissue types that were more strongly associated with SCZ than across disorders – we performed stepwise conditional analyses on the remaining 14 gene sets and 11 tissue types. In the first step, we tested the independence of the identified tissue types using a forward selection procedure. This revealed that the brain tissue-type associations were strongly interdependent (online Supplementary Table S3), and only cerebellum and frontal cortex reflected distinct signals.

**Table 2.** Overlap in genes between 14 gene sets significantly associated across disorders

	Highly-brain-expressed genes (BrainSpan)	FMRP targets, Darnell *et al*. (2011)	GO high voltage gated calcium channel activity	GO postsynapse	MIR137 targetscan v6.2, Lewis *et al*. (2005)	GO modulation of synaptic transmission	GO synapse part	GO synapse	GO neuron spine	GO membrane depolarization during cardiac muscle cell action potential	GO gamma aminobutyric acid signaling pathway	GO transcription factor activity RNA polymerase II core promoter sequence specific	GO voltage gated calcium channel complex	GO excitatory synapse
Highly-brain-expressed genes (BrainSpan)	5610													
FMRP targets, Darnell *et al*. (2011)	558	782												
GO high voltage gated calcium channel activity	2	5	11											
GO postsynapse	230	90	1	378										
MIR137 targetscan v6.2, Lewis *et al*. (2005)	452	104	3	43	1113									
GO modulation of synaptic transmission	177	72	1	99	43	301								
GO synapse part	375	140	3	378	70	152	610							
GO synapse	450	164	4	378	81	172	610	754						
GO neuron spine	84	35	0	119	14	47	119	120	121					
GO membrane depolarization during cardiac muscle cell action potential	6	4	3	5	6	1	5	5	3	14				
GO gamma aminobutyric acid signaling pathway	12	3	2	16	2	5	17	18	1	0	23			
GO transcription factor activity RNA polymerase II core promoter sequence specific	3	0	0	1	2	2	1	1	0	0	0	14		
GO voltage gated calcium channel complex	8	8	11	4	7	8	6	7	2	5	2	0	40	
GO excitatory synapse	132	66	1	190	28	66	195	197	55	1	0	0	4	197

Each cell includes the number of genes that overlap between two gene sets. Cells on the diagonal show the total number of genes in the gene set. The 11 GO gene sets are extracted from the Gene Ontology database. The other three gene sets are expert curated.

Second, we corrected the gene sets for global effects of confounding caused by the average gene expression levels, and the tissue types cerebellum and frontal cortex that were identified in the previous step. The highly-brain-expressed gene set was captured for the most part by the gene expression levels as expected. The other gene-set associations showed minor to moderate reductions, though still strongly associated across disorders (online Supplementary Table S2), indicating that the associations of the identified gene sets are not merely because they comprise genes expressed in the brain.

Third, we applied the forward selection procedure to the gene sets to shed light on the relations between their associations. We excluded the highly-brain-expressed gene set from this step as gene expression levels are already captured by conditioning on the average, cerebellum and frontal cortex tissue expression levels included in the previous step. The eight gene sets *FMRP targets* (Darnell *et al*., 2011), *GO high voltage gated calcium channel activity*, *GO postsynapse*, *MIR137 targets* (Lewis *et al*., 2005), *GO modulation of synaptic transmission*, *GO membrane depolarization during cardiac muscle cell action potential*, *GO gamma aminobutyric acid signaling pathway*, and *GO transcription factor activity RNA polymerase II core promoter sequence specific* showed independent effects. However, the associations with the gene sets *GO synapse part*, *GO synapse*, *GO neuron spine*, *GO voltage gated calcium channel complex*, and *GO excitatory synapse* could fully be accounted for by the other identified gene sets. The large overlap of genes in these sets with the other identified gene sets (Table 2) clearly represents a shared association signal.

## Discussion

The current gene-set analyses revealed various new sets of genes – in particular related to the synapse and neuronal functions – and gene-expression profiles of multiple brain tissues that play a role in shared genetic risk across five psychiatric disorders. The most strongly associated gene set was the highly-brain-expressed genes, which has previously been related to ASD (Pinto *et al*., 2014). However, as this gene set contains over 5000 genes with many different functions, this observation particularly confirms the polygenic nature of psychiatric disorders and its association with brain processes. This finding is in concordance with our tissue-type analysis, which showed the importance of gene expression of brain tissues for psychiatric disorders. Gene expression profiles of the cerebellum showed the strongest association, which confirms studies reporting cerebellar dysfunction in various psychiatric disorders (Phillips *et al*., 2015). Our finding of an additional effect of expression profiles of the frontal cortex is supported by observations that dysfunction of this region and related networks underlie cognitive and behavioral disturbances in psychiatric disorders (Fornito *et al*., 2015).

In addition, we identified multiple gene sets related to the synapse, which aligns with synaptic functions of several identified genes for multiple individual psychiatric disorders (Schizophrenia Working Group of the Psychiatric Genomics Consortium, 2014; Wray *et al*., 2018; Demontis *et al*., 2019; Grove *et al*., 2019; Stahl *et al*., 2019). Three of these gene sets point to a specific role of calcium channels, a well-established mechanism related to SCZ (Ripke *et al*., 2013; Schizophrenia Working Group of the Psychiatric Genomics Consortium, 2014; Pardinas *et al*., 2018) and suggested for BP and MDD as well (Cross-Disorder Group of the Psychiatric Genomics Consortium, 2013*b*; Wray *et al*., 2018). A common role across additional disorders is further supported by a cross-disorder genome-wide meta-analysis reporting genes related to the functioning of these channels (Schork *et al*., 2019). We also replicated a cross-disorder role for the postsynapse, although our findings do not support the previously reported role of histone and immune pathways (The Network and Pathway Analysis Subgroup of the Psychiatric Genomics Consortium, 2015). The target genes of MIR137, a microRNA that is one of the best replicated genetic risk factors for SCZ (Cross-Disorder Group of the Psychiatric Genomics Consortium, 2013*b*; Ripke *et al*., 2013; Pardinas *et al*., 2018), have not been implicated yet in other disorders and we now show that alterations in this network of genes are likely also involved in other psychiatric disorders. Multiple studies have reported the involvement of MIR137 in synaptic function, by regulating synaptogenesis, synapse maturation and synaptic transmission (Strazisar *et al*., 2014; Verma *et al*., 2015; He *et al*., 2018). Furthermore, our results suggest a shared role for FMRP targets which have previously been related to SCZ and ASD based on CNVs (Pinto *et al*., 2014; Szatkiewicz *et al*., 2014), *de novo* mutations (Iossifov *et al*., 2012; Fromer *et al*., 2014), rare variants (Purcell *et al*., 2014), and common variants (Schizophrenia Working Group of the Psychiatric Genomics Consortium, 2014; Jansen *et al*., 2017; Pardinas *et al*., 2018). FMRP is an RNA-binding protein involved in the regulation of translation. The binding transcripts code mainly for postsynaptic proteins (Darnell *et al*., 2011), and loss of FMRP results in widespread deficits in synaptic plasticity (Darnell and Klann, 2013). Taken together, all identified gene sets converge to an important contribution of communication between neurons, which is supported by the implication of a more common role of altered cortical connectivity in psychiatric disorders (Fornito *et al*., 2015).

Of note, the biological annotations of gene sets comprise a complex and challenging process, e.g., due to the multiple functions of many genes and incomplete knowledge. The construction of gene sets is in general based on different approaches such as shared cellular mechanism, co-expression patterns, protein-protein interaction, or co-localization. Hence, sets of genes may be based upon different inclusion criteria, creating an overlap between gene sets, as also illustrated by the current study. Clearly, it is important to recognize the impact of particular annotations on gene-set analysis results and their biological interpretation.

To address this issue of confounding and redundancy in gene sets, we applied conditional analyses. This provided insight in how different gene-set associations relate to each other, and whether identified functions may not be biologically relevant to the disorders but rather induced by confounding factors (de Leeuw *et al*., 2018). Brain-specific gene expression could be such a general confounder for our identified synaptic and neuronal gene sets, but the conditional analyses demonstrated that most of their associated signals were independent of brain expression levels. The conditional analyses between the identified gene sets revealed that part of these gene-set associations is not independent, which might be induced by a more extensive underlying function. Nevertheless, several independent associations suggest that multiple synaptic mechanisms are contributing risk factors for psychiatric disorders. These mechanisms may serve as starting points for future functional studies to disentangle their relation to psychiatric disorders, and potentially provide a first resource for the identification of drug targets and for drug repositioning (Breen *et al*., 2016).

Our cross-disorder gene-set and gene-property analyses are built on a meta-analysis of the gene-based associations with the individual disorders, therefore possible opposite effects of genetic variants are not taken into account. To explore if genetic variants are related to multiple disorders but with opposite effects, we performed an SNP-based meta-analysis of the five disorders and conducted a gene-set and gene-property analysis based on those results. In this analysis, genetic variants with opposite effects across disorders are cancelled out. Although these results showed strong correlations with the original analysis, we detected differences in association strength that point to partial differences in direction of SNP effects between the disorders for most identified gene sets. Interestingly, the effects on calcium channel activity are unidirectional across disorders. The outcome of different effects across disorders is supported by the recent finding that the highly correlated disorders SCZ and BD are differentiated by several genetic loci with opposite directions of effects (Bipolar Disorder and Schizophrenia Working Group of the Psychiatric Genomics Consortium, 2018). It has indeed been shown that in addition to genetic variants with effects on a general dimension of cross-disorder liability, specific variants uniquely differentiate between psychiatric disorders (Grotzinger *et al*., 2019). Furthermore, the general cross-disorder liability could reflect biological mechanisms that are related to specific overlapping symptoms, e.g. sleep disturbances, depressive symptoms and cognitive problems. This hypothesis is supported by the finding that polygenic components underlie multiple symptom dimensions of SCZ and BD (Bipolar Disorder and Schizophrenia Working Group of the Psychiatric Genomics Consortium, 2018). Exploring the biological mechanisms that may drive specific symptoms across disorders is therefore required to further advance our understanding of the complexity of the genetic overlap. Moreover, the identification of these mechanisms may help to develop individual-centered therapy driven by symptoms instead of general disorders. Moving the focus from dichotomies to the level of the individual is required to advance precision medicine (Senn, 2018).

We note that the associations of our identified gene sets were to a large extent driven by SCZ. This is in line with previous studies that reported multiple gene sets associated with SCZ (Ripke *et al*., 2013; Schizophrenia Working Group of the Psychiatric Genomics Consortium, 2014; Pardinas *et al*., 2018). Yet, hardly any gene sets have been detected for other psychiatric disorders despite the recent successes in identifying many genetic loci for multiple disorders that resulted from the fast increase in sample sizes that approach, or even exceed, the sample sizes of SCZ studies. This suggests that less successful findings for disorders such as MDD are unlikely a result of less statistical power. One possible explanation is that the identified gene sets of the current study have a true stronger effect on SCZ. One could also speculate that that SCZ has a different genetic architecture, or is less genetically heterogeneous compared to other disorders, but future studies are needed to address these issues.

In conclusion, the current study provides novel evidence for shared biological mechanisms that act across psychiatric disorders based on gene-set and gene-property analyses. We showed that several gene sets that previously only had been associated with individual disorders also play a role in a broader range of psychiatric disorders, supporting the view of a common pathogenesis across disorders. This indicates that the genetic overlap between disorders is not randomly distributed, but can be explained by specific biological mechanisms. The strongest evidence in our results was for the involvement of synaptic functions, and gene expression profiles of the cerebellum and frontal cortex. The genetic data collection of additional psychiatric disorders is rapidly increasing and will make it possible to extend our analyses to other disorders in the near future. Understanding the shared biological mechanisms between psychiatric disorders may provide a hint towards a general vulnerability for multiple psychiatric disorders, and could result in potential treatment for a broad spectrum of psychiatric disorders.

## Supplementary material

For supplementary material accompanying this paper visit http://dx.doi.org/10.1017/S0033291719001776. click here to view supplementary material
