# Supplementary material for: Synaptic and brain-expressed gene sets relate to the shared genetic risk across five psychiatric disorders
Source: Psychol Med. 2019 Jul 22;50(10):1695–705. doi: 10.1017/S0033291719001776 (PMC7408577; doi:10.1017/S0033291719001776)
Supplement: Supplementary file 1 [file S0033291719001776sup.zip › S0033291719001776sup001.pdf]

Synaptic and brain-expressed gene sets relate to the shared genetic risk across five psychiatric disorders

Anke R. Hammerschlag[1,2,3], Christiaan A. de Leeuw[1], Christel M. Middeldorp[2,3,4] and Tinca J.C. Polderman[1]

1 Department of Complex Trait Genetics, Center for Neurogenomics and Cognitive Research, Amsterdam Neuroscience, Vrije Universiteit Amsterdam, Amsterdam, the Netherlands
2 Child Health Research Centre, the University of Queensland, Brisbane, QLD, Australia
3 Department of Biological Psychology, Amsterdam Public Health, Vrije Universiteit Amsterdam, Amsterdam, the Netherlands
4 Child and Youth Mental Health Service, Children's Health Queensland Hospital and Health Service, Brisbane, QLD, Australia

SUPPLEMENTARY MATERIAL

Supplementary Methods

Minor influence of sample overlap on gene-set analysis

Sample overlap is an important concern in genetic cross-disorder analyses. Therefore, we corrected for sample overlap between the five disorders of this study. However, as explained below, in contrast to a gene based and SNP based analysis, the effect of sample overlap on ***gene-set analysis*** is limited.

For gene meta-analysis of K independent cohorts, under the null hypothesis that gene g is not associated with the phenotype the length K vector of Z-statistics for that gene Z_g has a $\mathrm{MVN}(0, I_K)$ distribution. These Z-statistics are combined as a weighted sum with the weight vector W_g, which in matrix notation can be written as $W_g^T Z_g$. This sum is distributed as $\mathrm{N}\left(0, W_g^T W_g\right)$, and therefore the meta-analysis Z-statistic $Z_{meta,g} = \frac{W_g^T Z_g}{\sqrt{W_g^T W_g}}$ has a standard normal distribution.

If there is sample overlap between the cohorts, then under the null hypothesis the Z-statistics will be correlated, such that $Z_g \sim \mathrm{MVN}\left(0, S_g\right)$ for some correlation matrix S_g. In this case, $W_g^T Z_g \sim \mathrm{N}\left(0, W_g^T S_g W_g\right)$ and $Z_{meta,g} = \frac{W_g^T Z_g}{\sqrt{W_g^T S_g W_g}}$.

In practice the sample overlap will need to be estimated from the SNP summary statistics using for example LD Score regression, and consequently there will only be a single estimated correlation matrix $\hat{S}$, which is used for all genes g. The weights are defined as $w_{g,j} = \sqrt{N_{g,j}}$, where $N_{g,j}$ is the sample size for the jth cohort for gene g. The sample size in a cohort can vary somewhat across genes because some SNPs have higher levels of missingness than others, or because the cohort itself represents a meta-analysis and not all of the underlying cohorts that meta-analysis was based on contained the same SNPs. In general this variation will be relative minor however, and therefore $W_g \approx W$ for all genes g.

Because of this, computing the vector Z_{meta} under the assumption $Z_g \sim \mathrm{MVN}(0, S)$ rather than $Z_g \sim \mathrm{MVN}(0, I_K)$ effectively scales the entire vector by the constant $\sqrt{\frac{W^T W}{W^T S W}}$. In the gene-set analysis Z_{meta} is used only as the dependent variable in a linear regression, and as such this scaling has no effect on the resulting *p* values.

Supplementary Tables

Supplementary Table 1. Overview of the 126 expert-curated gene sets included in the gene-set analysis.

Group of gene sets	*N* gene sets	Studied disorders	References	Source
Calcium signaling	2	SCZ, MDD	Müller *et al.* 2010; Ripke *et al.* 2013; Purcell *et al.* 2014; Pardinas *et al.* 2018; Wray *et al.* 2018	Authors of Ripke *et al.* 2013
FMRP interactors	3	ASD, SCZ, MDD	Darnell *et al.* 2011; Ascano *et al.* 2012; Iossifov *et al.* 2012; Szatkiewicz *et al.* 2014; Fromer *et al.* 2014; Pinto *et al.* 2014; Purcell *et al.* 2014; Schizophrenia Working Group of the Psychiatric Genomics Consortium 2014; Anney *et al.* 2017; Jansen *et al.* 2017; Wray *et al.* 2018; Pardinas *et al.* 2018	Authors of Purcell et al. 2014
Glia functional gene sets: oligodendrocytes, astrocytes, microglia (based on expression patterns)	79	SCZ, ASD	Goudriaan *et al.* 2014; Jansen *et al.* 2017	https://ctg.cncr.nl/software/genesets
Highly-brain-expressed genes (defined as log(RPKM[reads per kb per million reads]) > 4.5 in BrainSpan)	1	ASD	Pinto *et al.* 2014; Anney *et al.* 2017	Authors of Pinto *et al.* 2014
MiR-137 predicted targets (detected by TargetScan)	2	SCZ, MDD	Lewis *et al.* 2005; Schizophrenia Psychiatric Genome-Wide Association Study (GWAS) Consortium 2011; Ripke *et al.* 2013; Szatkiewicz *et al.* 2014; Wray *et al.* 2018	Authors of Ripke *et al.* 2013
Mitochondrial genes (human nuclear-encoded in MitoCarta)	1	SCZ, ASD	Pagliarini *et al.* 2008; Szatkiewicz *et al.* 2014; Jansen *et al.* 2017	https://www.broadinstitute.org/scientific-community/science/programs/metabolic-disease-program/publications/mitocarta/mitocarta-in-0
Protein-protein interaction networks derived from ASD *de novo* mutations	3	ASD	O'Roak *et al.* 2012; Purcell *et al.* 2014	Authors of Purcell *et al.* 2014
Synaptic functional gene sets (based on cellular function)	18	SCZ, ADHD, ASD	Ruano *et al.* 2010; Lips *et al.* 2012; Hammerschlag *et al.* 2014; Jansen *et al.* 2017	https://ctg.cncr.nl/software/genesets

Synaptic protein complexes and generic subcellular components (based on proteomics data sets)	17	SCZ, MDD	Kirov *et al.* 2012; Fromer *et al.* 2014; Purcell *et al.* 2014; Szatkiewicz *et al.* 2014; Pardinas *et al.* 2018; Wray *et al.* 2018	Authors of Purcell *et al*. 2014

FMRP, Fragile X mental retardation protein; RPKM, reads per kb per million reads; SCZ, schizophrenia; MDD, major depressive disorder; ASD, autism spectrum disorder; ADHD, attention-deficit/hyperactive disorder.

Supplementary Table 2. Gene-set associations across five psychiatric disorders and gene-set associations in post hoc analyses.

Supplementary excel file.

Supplementary Table 3. Tissue-type associations across five psychiatric disorders and tissue-type associations in post hoc analyses

Supplementary excel file.

Supplementary References

Anney RJL, Ripke S, Anttila V, Grove J, Holmans P, Huang H, Klei L, Lee PH, Medland SE, Neale B, Robinson E, Weiss LA, Zwaigenbaum L, Yu TW, Wittemeyer K, Willsey AJ, Wijsman EM, Werge T, Wassink TH, Waltes R, Walsh CA, Wallace S, Vorstman JAS, Vieland VJ, Vicente AM, vanEngeland H, Tsang K, Thompson AP, Szatmari P, Svantesson O, Steinberg S, Stefansson K, Stefansson H, State MW, Soorya L, Silagadze T, Scherer SW, Schellenberg GD, Sandin S, Sanders SJ, Saemundsen E, Rouleau GA, Rogé B, Roeder K, Roberts W, Reichert J, Reichenberg A, Rehnström K, Regan R, Poustka F, Poultney CS, Piven J, Pinto D, Pericak-Vance MA, Pejovic-Milovancevic M, Pedersen MG, Pedersen CB, Paterson AD, Parr JR, Pagnamenta AT, Oliveira G, Nurnberger JI, Nordentoft M, Murtha MT, Mouga S, Mortensen PB, Mors O, Morrow EM, Moreno-De-Luca D, Monaco AP, Minshew N, Merikangas A, McMahon WM, McGrew SG, Mattheisen M, Martsenkovsky I, Martin DM, Mane SM, Magnusson P, Magalhaes T, Maestrini E, Lowe JK, Lord C, Levitt P, Martin CL, Ledbetter DH, Leboyer M, LeCouteur AS, Ladd-Acosta C, Kolevzon A, Klauck SM, Jacob S, Iliadou B, Hultman CM, Hougaard DM, Hertz-Picciotto I, Hendren R, Hansen CS, et al. (2017). Meta-analysis of GWAS of over 16,000 individuals with autism spectrum disorder highlights a novel locus at 10q24.32 and a significant overlap with schizophrenia. *Molecular Autism* **8**, 21.

Ascano M, Mukherjee N, Bandaru P, Miller JB, Nusbaum JD, Corcoran DL, Langlois C, Munschauer M, Dewell S, Hafner M, Williams Z, Ohler U, Tuschl T (2012). FMRP targets distinct mRNA sequence elements to regulate protein expression. *Nature* **492**, 382–386.

Darnell JC, Van Driesche SJ, Zhang C, Hung KYS, Mele A, Fraser CE, Stone EF, Chen C, Fak JJ, Chi SW, Licatalosi DD, Richter JD, Darnell RB (2011). FMRP stalls ribosomal translocation on mRNAs linked to synaptic function and autism. *Cell* **146**, 247–261.

Fromer M, Pocklington AJ, Kavanagh DH, Williams HJ, Dwyer S, Gormley P, Georgieva L, Rees E, Palta P, Ruderfer DM, Carrera N, Humphreys I, Johnson JS, Roussos P, Barker DD, Banks E, Milanova V, Grant SG, Hannon E, Rose S a, Chambert K, Mahajan M, Scolnick EM, Moran JL, Kirov G, Palotie A, McCarroll S a, Holmans P, Sklar P, Owen MJ, Purcell SM, O'Donovan MC (2014). De novo mutations in schizophrenia implicate synaptic networks. *Nature* **506**, 179–184.

Goudriaan A, De Leeuw C, Ripke S, Hultman CM, Sklar P, Sullivan PF, Smit AB, Posthuma D, Verheijen MHG (2014). Specific glial functions contribute to Schizophrenia susceptibility. *Schizophrenia Bulletin* **40**, 925–935.

Hammerschlag AR, Polderman TJC, de Leeuw C, Tiemeier H, White T, Smit AB, Verhage M, Posthuma D (2014). Functional Gene-Set Analysis Does Not Support a Major Role for Synaptic Function in Attention Deficit/Hyperactivity Disorder (ADHD). *Genes* **5**, 604–614.

Iossifov I, Ronemus M, Levy D, Wang Z, Hakker I, Rosenbaum J, Yamrom B, Lee YH, Narzisi G, Leotta A, Kendall J, Grabowska E, Ma B, Marks S, Rodgers L, Stepansky A, Troge J, Andrews P, Bekritsky M, Pradhan K, Ghiban E, Kramer M, Parla J, Demeter R, Fulton LL, Fulton RS, Magrini VJ, Ye K, Darnell JC, Darnell RB, Mardis ER, Wilson RK, Schatz MC, McCombie RW, Wigler M (2012). De novo gene disruptions in children on the autistic spectrum. *Neuron* **74**, 285–299.

Jansen A, Dieleman GC, Smit AB, Verhage M, Verhulst FC, Polderman TJC, Posthuma D (2017). Gene-set analysis shows association between FMRP targets and autism spectrum disorder. *European Journal of Human Genetics* **25**, 1–6.

Kirov G, Pocklington A, Holmans PA, Ivanov DK, Ikeda M, Ruderfer D, Moran J, Chambert K, Toncheva D, Georgieva L, Grozeva DV, Fjodorova M, Wollerton RL, Rees E, Nikolova I, van de Lagemaat LN, Bayés À, Fernandez E, Olason PI, Böttcher Y, Komiyama NH, Collins MO, Choudhary J, Stefansson K, Stefansson H, Grant SGN, Purcell S, Sklar P, O'Donovan MC, Owen MJ (2012). De novo CNV analysis implicates specific abnormalities of postsynaptic signalling complexes in the pathogenesis

of schizophrenia. *Molecular Psychiatry* **17**, 142–153.
Lewis BP, Burge CB, Bartel DP (2005). Conserved seed pairing, often flanked by adenosines, indicates that thousands of human genes are microRNA targets. *Cell* **120**, 15–20.
Lips ES, Cornelisse LN, Toonen RF, Min JL, Hultman CM, Holmans P a, O'Donovan MC, Purcell SM, Smit a B, Verhage M, Sullivan PF, Visscher PM, Posthuma D (2012). Functional gene group analysis identifies synaptic gene groups as risk factor for schizophrenia. *Molecular Psychiatry* **17**, 996–1006.
Müller CS, Haupt A, Bildl W, Schindler J, Knaus H-G, Meissner M, Rammner B, Striessnig J, Flockerzi V, Fakler B, Schulte U (2010). Quantitative proteomics of the Cav2 channel nano-environments in the mammalian brain. *Proceedings of the National Academy of Sciences of the United States of America* **107**, 14950–14957.
O'Roak BJ, Vives L, Girirajan S, Karakoc E, Krumm N, Coe BP, Levy R, Ko A, Lee C, Smith JD, Turner EH, Stanaway IB, Vernot B, Malig M, Baker C, Reilly B, Akey JM, Borenstein E, Rieder MJ, Nickerson D a., Bernier R, Shendure J, Eichler EE (2012). Sporadic autism exomes reveal a highly interconnected protein network of de novo mutations. *Nature* **485**, 246–250.
Pagliarini DJ, Calvo SE, Chang B, Sheth S a., Vafai SB, Ong SE, Walford G a., Sugiana C, Boneh A, Chen WK, Hill DE, Vidal M, Evans JG, Thorburn DR, Carr S a., Mootha VK (2008). A Mitochondrial Protein Compendium Elucidates Complex I Disease Biology. *Cell* **134**, 112–123.
Pardinas AF, Holmans P, Pocklington AJ, Escott-Price V, Ripke S, Carrera N, Legge SE, Bishop S, Cameron D, Hamshere ML, Han J, Hubbard L, Lynham A, Mantripragada K, Rees E, MacCabe JH, McCarroll SA, Baune BT, Breen G, Byrne EM, Dannlowski U, Eley TC, Hayward C, Martin NG, McIntosh AM, Plomin R, Porteous DJ, Wray NR, Caballero A, Geschwind DH, Huckins LM, Ruderfer DM, Santiago E, Sklar P, Stahl EA, Won H, Agerbo E, Als TD, Andreassen OA, Baekvad-Hansen M, Mortensen PB, Pedersen CB, Borglum AD, Bybjerg-Grauholm J, Djurovic S, Durmishi N, Pedersen MG, Golimbet V, Grove J, Hougaard DM, Mattheisen M, Molden E, Mors O, Nordentoft M, Pejovic-Milovancevic M, Sigurdsson E, Silagadze T, Hansen CS, Stefansson K, Stefansson H, Steinberg S, Tosato S, Werge T, Collier DA, Rujescu D, Kirov G, Owen MJ, O'Donovan MC, Walters JTR (2018). Common schizophrenia alleles are enriched in mutation-intolerant genes and in regions under strong background selection. *Nature Genetics* **50**, 381–389.
Pinto D, Delaby E, Merico D, Barbosa M, Merikangas A, Klei L, Thiruvahindrapuram B, Xu X, Ziman R, Wang Z, Vorstman J a S, Thompson A, Regan R, Pilorge M, Pellecchia G, Pagnamenta AT, Oliveira B, Marshall CR, Magalhaes TR, Lowe JK, Howe JL, Griswold AJ, Gilbert J, Duketis E, Dombroski B a., De Jonge M V., Cuccaro M, Crawford EL, Correia CT, Conroy J, Conceição IC, Chiocchetti AG, Casey JP, Cai G, Cabrol C, Bolshakova N, Bacchelli E, Anney R, Gallinger S, Cotterchio M, Casey G, Zwaigenbaum L, Wittemeyer K, Wing K, Wallace S, Van Engeland H, Tryfon A, Thomson S, Soorya L, Rogé B, Roberts W, Poustka F, Mouga S, Minshew N, McInnes LA, McGrew SG, Lord C, Leboyer M, Le Couteur AS, Kolevzon A, Jiménez González P, Jacob S, Holt R, Guter S, Green J, Green A, Gillberg C, Fernandez B a., Duque F, Delorme R, Dawson G, Chaste P, Café C, Brennan S, Bourgeron T, Bolton PF, Bölte S, Bernier R, Baird G, Bailey AJ, Anagnostou E, Almeida J, Wijsman EM, Vieland VJ, Vicente AM, Schellenberg GD, Pericak-Vance M, Paterson AD, Parr JR, Oliveira G, Nurnberger JI, Monaco AP, Maestrini E, Klauck SM, Hakonarson H, Haines JL, Geschwind DH, Freitag CM, et al. (2014). Convergence of genes and cellular pathways dysregulated in autism spectrum disorders. *American Journal of Human Genetics* **94**, 677–694.
Purcell SM, Moran JL, Fromer M, Ruderfer D, Solovieff N, Roussos P, O'Dushlaine C, Chambert K, Bergen SE, Kähler A, Duncan L, Stahl E, Genovese G, Fernández E, Collins MO, Komiyama NH, Choudhary JS, Magnusson PKE, Banks E, Shakir K, Garimella K, Fennell T, DePristo M, Grant SGN, Haggarty SJ, Gabriel S, Scolnick EM, Lander ES, Hultman CM, Sullivan PF, McCarroll S a, Sklar P (2014). A polygenic burden of rare disruptive mutations in schizophrenia. *Nature* **506**, 185–190.
Ripke S, O'Dushlaine C, Chambert K, Moran JL, Kahler AK, Akterin S, Bergen SE, Collins AL, Crowley JJ,

Fromer M, Kim Y, Lee SH, Magnusson PKE, Sanchez N, Stahl EA, Williams S, Wray NR, Xia K, Bettella F, Borglum AD, Bulik-Sullivan BK, Cormican P, Craddock N, de Leeuw C, Durmishi N, Gill M, Golimbet V, Hamshere ML, Holmans P, Hougaard DM, Kendler KS, Lin K, Morris DW, Mors O, Mortensen PB, Neale BM, O'Neill FA, Owen MJ, Milovancevic MP, Posthuma D, Powell J, Richards AL, Riley BP, Ruderfer D, Rujescu D, Sigurdsson E, Silagadze T, Smit AB, Stefansson H, Steinberg S, Suvisaari J, Tosato S, Verhage M, Walters JT, Levinson DF, Gejman P V, Kendler KS, Laurent C, Mowry BJ, O'Donovan MC, Owen MJ, Pulver AE, Riley BP, Schwab SG, Wildenauer DB, Dudbridge F, Holmans P, Shi J, Albus M, Alexander M, Campion D, Cohen D, Dikeos D, Duan J, Eichhammer P, Godard S, Hansen M, Lerer FB, Liang K-Y, Maier W, Mallet J, Nertney DA, Nestadt G, Norton N, O'Neill FA, Papadimitriou GN, Ribble R, Sanders AR, Silverman JM, Walsh D, Williams NM, Wormley B, Arranz MJ, Bakker S, Bender S, Bramon E, Collier D, Crespo-Facorro B, et al. (2013). Genome-wide association analysis identifies 13 new risk loci for schizophrenia. *Nature Genetics* **45**, 1150–1159.

Ruano D, Abecasis GR, Glaser B, Lips ES, Cornelisse LN, de Jong APH, Evans DM, Davey Smith G, Timpson NJ, Smit AB, Heutink P, Verhage M, Posthuma D (2010). Functional gene group analysis reveals a role of synaptic heterotrimeric G proteins in cognitive ability. *American Journal of Human Genetics* **86**, 113–125.

Schizophrenia Psychiatric Genome-Wide Association Study (GWAS) Consortium (2011). Genome-wide association study identifies five new schizophrenia loci. *Nature Genetics* **43**, 969–976.

Schizophrenia Working Group of the Psychiatric Genomics Consortium (2014). Biological insights from 108 schizophrenia-associated genetic loci. *Nature* **511**, 421–427.

Szatkiewicz JP, O'Dushlaine C, Chen G, Chambert K, Moran JL, Neale BM, Fromer M, Ruderfer D, Akterin S, Bergen SE, Kähler A, Magnusson PKE, Kim Y, Crowley JJ, Rees E, Kirov G, O'Donovan MC, Owen MJ, Walters J, Scolnick E, Sklar P, Purcell S, Hultman CM, McCarroll S a, Sullivan PF (2014). Copy number variation in schizophrenia in Sweden. *Molecular Psychiatry* **19**, 762–773.

Wray NR, Ripke S, Mattheisen M, Trzaskowski M, Byrne EM, Abdellaoui A, Adams MJ, Agerbo E, Air TM, Andlauer TMF, Bacanu S-A, Baekvad-Hansen M, Beekman AFT, Bigdeli TB, Binder EB, Blackwood DRH, Bryois J, Buttenschon HN, Bybjerg-Grauholm J, Cai N, Castelao E, Christensen JH, Clarke T-K, Coleman JIR, Colodro-Conde L, Couvy-Duchesne B, Craddock N, Crawford GE, Crowley CA, Dashti HS, Davies G, Deary IJ, Degenhardt F, Derks EM, Direk N, Dolan C V, Dunn EC, Eley TC, Eriksson N, Escott-Price V, Kiadeh FHF, Finucane HK, Forstner AJ, Frank J, Gaspar HA, Gill M, Giusti-Rodriguez P, Goes FS, Gordon SD, Grove J, Hall LS, Hannon E, Hansen CS, Hansen TF, Herms S, Hickie IB, Hoffmann P, Homuth G, Horn C, Hottenga J-J, Hougaard DM, Hu M, Hyde CL, Ising M, Jansen R, Jin F, Jorgenson E, Knowles JA, Kohane IS, Kraft J, Kretzschmar WW, Krogh J, Kutalik Z, Lane JM, Li Y, Li Y, Lind PA, Liu X, Lu L, MacIntyre DJ, MacKinnon DF, Maier RM, Maier W, Marchini J, Mbarek H, McGrath P, McGuffin P, Medland SE, Mehta D, Middeldorp CM, Mihailov E, Milaneschi Y, Milani L, Mill J, Mondimore FM, Montgomery GW, Mostafavi S, Mullins N, et al. (2018). Genome-wide association analyses identify 44 risk variants and refine the genetic architecture of major depression. *Nature Genetics* **50**, 668–681.
